# Diagnostic utility of C-kit protein (CD117) expression in differentiating adenoid cystic carcinoma and polymorphous low grade Adenocarcinoma

**DOI:** 10.12669/pjms.336.13373

**Published:** 2017

**Authors:** Hassan Tariq, Saba Anjum, Hafeez ud Din, Farhan Akhtar

**Affiliations:** 1 Dr. Hassan Tariq, MBBS. Department of Histopathology, Armed Forces Institute of Pathology, PNS Shifa, Rawalpindi, Pakistan; 2 Dr. Saba Farrukh, MBBS. Department of Histopathology, Armed Forces Institute of Pathology, PNS Shifa, Rawalpindi, Pakistan; 3 Dr. Hafeez ud Din, FCPS. Assistant Professor, Department of Histopathology, Armed Forces Institute of Pathology, PNS Shifa, Rawalpindi, Pakistan; 4 Dr. Farhan Akhtar, FCPS. Assistant Professor Department of Histopathology, Armed Forces Institute of Pathology, PNS Shifa, Rawalpindi, Pakistan

**Keywords:** Adenoid cystic carcinoma, Polymorphous low-grade adenocarcinoma, C-kit, Salivary gland neoplasm

## Abstract

**Background & Objective:**

To evaluate usefulness of immunohistochemical marker C-kit (CD117) in differentiating Adenoid cystic carcinoma (AdCC) from Polymorphous low-grade adenocarcinoma (PLGA) in patients of salivary gland carcinomas. AdCC is a malignant salivary gland neoplasm with poor prognosis. PLGA is a salivary gland malignancy with indolent growth pattern. Differentiating between the two entities is a diagnostic challenge. We evaluated the role of C-kit in differentiating the two.

**Methods:**

This is a Cross sectional study. Samples of 19 tumors including 12 AdCC and 4 PLGA was evaluated at Department of Histopathology, Armed Forces Institute of Pathology, Rawalpindi from December 2015 to August 2016. Immunohistochemical techniques were used to analyze the level of c-kit expression in AdCC (n = 12), polymorphous low-grade adenocarcinoma (PLGA) (n = 6). Samples were stained using monoclonal antibody against C-kit. Statistical analysis of the data was done using SPSS version 21.

**Results:**

Strong diffuse cytoplasmic reactivity was observed in more than 50% of the tumor cells of AdCC whereas less than 20% of cells showed negative to weak positivity in PLGA. Hence, the difference in the expression of c-kit between AdCC and PLGA was statistically significant (p value <0.002).

**Conclusions:**

CD117 expression itself can be used as a marker in differential diagnosis of salivary gland neoplasms. However, the percentage of the CD117 immunoreactive cells and the staining intensities appeared to be important factors in distinguishing AdCC from PLGA.

## INTRODUCTION

CD117 (c-kit proto-oncogene) is a Type-III transmembrane receptor tyrosine Kinase that is encoded by the c-kit gene which is placed on the human chromosome segment 4q11.^1^,^2^ C-kit leads to activation of specific intracellular signal transduction cascades and thus plays a key part in normal differentiation and growth of different cell types.^3^-^5^

The role for C-kit and its mutant forms in carcinogenesis has been implicated in a number of human neoplasms including gastrointestinal stromal tumors, ovarian cancer like (dysgerminoma), acute myeloblastic leukemia, small and non-small lung cancers, testicular germ cell tumors like (seminoma), and malignant melanoma. These tumors show an overexpression of C-kit.^6^-^10^

Adenoid cystic carcinoma (AdCC) accounts for approximately 10% of all malignant salivary gland tumors. Its peak incidence is 40 to 60 year with a slight female preponderance. It is usually found in minor salivary glands of the mouth, particularly the palate, and the upper aerodigestive tract. Tumor is characterized by indolent course, a high rate of metastasis with late onset and low rate of long term survival.

PLGA is found almost exclusively in minor glands and accounts for about a quarter of the malignant tumors in these sites. It mostly presents in patients aged 50 to 70 years, with a female predominance. The most common site is the palate (60%), especially at the junction of the hard and soft palates. Tumors usually form a slow growing mass with excellent prognosis and low rate of local recurrences. Conservative surgery is the treatment of choice. Since AdCC exhibits invasive behavior, differentiating this tumor from PLGA that share similar histological features in small biopsies is critical.^11^,^12^

Currently, there is little information on the altered expression of C-kit in salivary gland tumors, which is mainly limited to AdCC and PLGA. Furthermore, recent studies about using C-kit for distinguishing AdCC from other salivary gland tumors are controversial. The purpose of this work is to determine the usefulness of immunohistochemical expression of CD117 in differentiating adenoid cystic carcinoma and polymorphous low grade adenocarcinoma and to evaluate the application of C-kit as a marker in the diagnosis of AdCC.

## METHODS

Ethical Committee of AFIP approved this study. Keeping confidence interval at 5% and power of the test at 80% sample size was calculated from WHO calculator. We obtained paraffin-embedded tissue blocks of 19 of understudy tumors including 12 AdCC and 4 PLGA from Department of Histopathology, Armed Forces Institute of Pathology, Rawalpindi from December 2015 to August 2016.

### Inclusion criteria

All specimens of adenoid cystic carcinoma and polymorphous low grade carcinoma of salivary gland detected by immunohistochemistry, and routine histopathology irrespective of age of patient, histological type and grade of the tumor, were included.

### Exclusion criteria

Poorly fixed specimens and specimens with scanty tumour tissue were be included.

Immunohistochemical assays for CD117 will be done by using BioSB kit as per the manufacturer’s guidelines. Immunohistochemistry results were interpreted on high power field objective. The results were verified by second opinion through intradepartmental consultation with various consultants to minimize bias. Statistical analysis were done by using SPSS calculator version 21. Frequency and percentage were calculated for qualitative variable like result outcome of CD117 by immunohistochemistry. Post stratification chi-square test was applied. P value < 0.002 was considered significant. The paraffin-embedded tissue blocks were sliced into three-micrometer sections for routine histological and subsequent immunohistochemical examinations. Diagnosis of the AdCC, PLGA was based on a combination of histological, immunological and clinical examination of the hematoxylin-and-eosin-stained tissue sections.

Immunohistochemistry results were interpreted on light microscope using high power field objective. Cytoplasmic CD117 expression was regarded as positive staining. Results were verified by other consultants to decrease the biases. Percentage and intensity of cytoplasmic staining of cells was independently reported by consultants and consensus was reached. The extent of CD117 expression for tumor was scored semi-quantitatively as per Table-I.

**Table-I:** Scoring of expression of CD117 in terms of intensity of Cytoplasmic Staining and percentage of cells stained.^17^,^18^

*Intensity of Cytoplasmic staining*	*Percentage of cells %*	*Score*	*Result*
Negative/weak	Less than 20	0	Negative/Weak
Weak/Moderate	20-50	1+	Moderate positive
Moderate/Strong	More than 50	2+	Strong positive
Intensity of Cytoplasmic staining	Percentage of cells %	Score	Result
Negative/weak	Less than 20	0	Negative/Weak
Weak/Moderate	20-50	1+	Moderate positive
Moderate/Strong	More than 50	2+	Strong positive

## RESULTS

Expression of CD117 was graded semi quantitatively in terms of intensity of cytoplasmic staining and percentage of cells stained and were graded as 0.1+ and 2+ as per Table-I. In case of approximately 90% of AdCC samples, more than 58% of the cells exhibited strong 2+ reactivity for C-kit, whereas 42% of the lesions showed moderate staining in about 20-50 % of the cells and were graded as 1+.CD117 showed mainly diffuse cytoplasmic staining with weak membranous component. In case of Plga 71% of cases showed no to weak staining and were graded as 0 whereas only 28 % of cases showed 1+ staining which was mainly cytoplasmic. The current study showed a significant difference (*P*< 0.002) in the expression of C-kit between the AdCC and PLGA tumors.

## DISCUSSION

The C-kit proto-oncogene protein (a transmembrane receptor Type-III tyrosine kinase) on binding to its ligand, stem cell factor begins a signal cascade that contributes to the growth and differentiation of multiple hematopoietic lineages. It shows structural homology to the receptors of platelet-derived growth factor, macrophage colony stimulating factor.^13^

Adenoid cystic carcinoma (AdCC) accounts for 10% of all malignant salivary gland tumors. It affects a wide age range with a peak incidence in 40- to 60-year-olds. There is a slight female preponderance. Minor glands of the mouth, particularly the palate and the upper aerodigestive tract account for about half of all cases. Other common sites include the parotid (21%), submandibular gland (5%), and sinonasal tract (11%). Tumor usually present as a slowly growing mass of long duration. It may be tender or painful, and cranial nerve lesions, particularly facial nerve palsy, may be the presenting feature. Tumors of minor glands often show ulceration of the overlying mucosa. It is characterized a high rate of metastasis with late onset and low rate of long-term survival.

**Table-II:** Expression of CD117 in AdCC.

*Score*	*Frequency*	*Percent*
1+	5	41.7
2+	7	58.3

Total	12	100.0

**Table-III:** Expression of CD117 in PLGA.

*Score of CD117*	*Frequency*	*Percent*
0	5	71.4
1+	2	28.6

Total	7	100.0

PLGA is found almost exclusively in minor glands and accounts for about a quarter of the malignant tumors in these sites. Most present in patients aged 50 to 70 years, with a female predominance (2:1). The most common site is the palate (60%), especially at the junction of the hard and soft palates. Other intraoral sites include the lip (particularly the upper), buccal mucosa, retromolar areas, and posterior third of the tongue. Rarely, tumors arise in the lacrimal gland, sinonasal tract, nasopharynx, and the upper and lower respiratory tracts. Tumors usually form a slow growing mass that may have been present for many years. Ulceration, bleeding, and pain are uncommon presenting features. Its prognosis is usually excellent with local recurrences in 9% to 17% of cases. Conservative surgery is the treatment of choice

Differentiating PLGA and AdCC on H&E morphology alone can be a difficult work especially if in case of biopsies dealing with minor salivary glands. Prognostic difference of the two is sufficiently different thus differentiating them becomes an important task of the pathologist. There is not much literature on the subject but several studies have shown a consistently strong expression of the C-kit protein in AdCC consistently.^14^,^15^ Penner et al. suggested C-kit to be a valuable tool for differentiating AdCC from PLGA. However Edwards et al. suggested that C-kit was not a useful marker for the said purpose.^12^ We decided to investigate the potential of this marker for the diagnosis of AdCC independently.

In addition to the role of C-kit in the diagnosis of AdCC, the relationship of C-kit expression with clinical findings was also evaluated by Lee et al. They reported that the expression of C-kit had no predictive value for recurrence and prognosis.^16^

**Table-IV:** Comparison of Intensity Scores of PLGA & AdCC.

*Tumour*	*Score*			*Total*

	*0+*	*1+*	*2+*	
PLGA	5	2	0	7
AdCC	0	5	7	12

Total	5	7	7	19

In this study, we explored C-kit protein expression in AdCC and PLGA. Comparing favorably with the previous literature almost all cases of AdCC exhibited strong expression of C-kit protein in this malignant neoplasm.^1^,^11^,^15^

AdCC classically has three predominant patterns. These are Tubular (well-differentiated or grade I), cribriform (moderately differentiated or grade II) and solid pattern (poorly differentiated or grade III). We encountered all the three types of patterns but predominantly cribriform pattern was seen. In the current study 58% of the lesions showed strong diffuse cytoplasmic staining in more than 50% of the cells whereas 42% of the lesions showed moderate staining in about 20-50% of the cells. These results are similar to the observations reported by Epivatianos et al.^17^, Andreadis et al.^18^ and Penner et al.^12^ The role of C-kit pathway in the pathogenesis of AdCC is a matter of debate as yet, but Oliveira *et al* suggested that C-kit does not have a direct oncogenic function in AdCC.^11^ The strong expression of C-kit in AdCC may suggest a role for C-kit inhibitors as potential therapeutic drugs for this tumor. Imatinib mesylate (Glivec) is a tyrosine kinase inhibitor (TKI) that inhibits both the platelet-derived growth factor receptor (PDGFR) and the C-kit receptor. In addition to the role of C-kit in the diagnosis of AdCC, the relationship of C-kit expression with clinical findings was also evaluated by Lee *et al*. They reported that the expression of C-kit had no predictive value for recurrence and prognosis.^16^

Histologically, PLGA is a malignant epithelial tumor characterized by *infiltrative* growth of cytologically uniform cells arranged in architecturally diverse patterns set within a characteristic stroma. Tumors are unencapsulated but well circumscribed. They incarcerate or entomb minor mucoserous glands and invade into the adjacent soft tissues. Perineural invasion is prominent and yields a “targetoid” appearance with the nerve that forms the nidus. In the current study expression of C-kit in PLGA was also assessed and it was found that 72% of the lesions showed no or weak cytoplasmic staining in less than 20% of the cells whereas only 28% of the lesions showed weak to moderate cytoplasmic staining in 20-50% of the cells which matched with the work of Andreadis et al.^18^ in the current subject.

## CONCLUSION

Based on the current work it is suggested that C-kit can aid in the differential diagnosis of lesion presenting a morphological confusion between AdCC and PLGA. This can aid considerably in the lesion of minor salivay gland where PLGA is common particularly in small biopsy specimens. The results could be more accurate if combined with the differential staining with other immunohistochemical markers like BCL-2 and Ki-67. Additionals investigation is required to characterize C-kit functional pathways in salivary gland tumors and to evaluate potential therapeutic effects of small molecule inhibitors of C-kit on these tumors.
